# Importance of Occlusion in Crown and Bridge-Work

**Published:** 1905-11-15

**Authors:** R. B. Howell


### IMPORTANCE OF OCCLUSION IN CROWN AND BRIDGE-WORK

BY R. B. HOWELL, D.D.S.

Read before the Michigan State Dental Society, July 12th, 1905.

The object of this paper is not to advance any new and outlined method relative to the application and preparation of crown and bridge work, but simply to emphasize the fact that "proper occlusion" is the most essential requirement for the usefulness of these artificial substitutes under consideration.

It has not been very long since the esthetic result was the important end in view for all dental operations. This is clearly shown in the practice of orthodontia. Parents visited the Orthodontist to "have their children's teeth straightened" because it improved their looks. They are now, however, learning that the true reason for correcting the irregularities is that they may have a better masticatory apparatus for the comminution of food, and thereby give to their descendants a better developed jaw and normally arranged teeth.

Crown and bridge work has had this same varied history, the important indication being at first the esthetic effect and now it is reverting to that of utility.

How unimportant is the idea of mere appearance is illustrated in the easy manner in which it is perverted. Every one remembers well the furious storm of gold crowns which raged in this country a few years ago, few mouths escaped the bombardment, its victims were numbered among all classes and conditions, from the girl who scrubs the floor to the lady who runs the home, and from the gentleman of leisure to the boy who shines his shoes. This storm finally abated, because of esthetic reasons, and the gold crown resumed its place among things which are useful rather than ornamental.

It is not the intention of the writer to eliminate all thought of the esthetic, because it is very essential and should be considered, in operations in all parts of the mouth; but it *is* the intention to make the following statement: When artificial substitutes are to be supplied in those places where true mastication takes place, namely, in the region of the bicuspids and molars, the idea of utility should predominate in the application and preparation of all crowns and bridges, thereby securing what might be called an artistic piece of work in every sense of the word.

There are a number of vital points which are essential to the construction of crowns and bridges, such as physiological relations, stress, approximal contact, method of attachment, etc., but the one to be considered in this paper is that of *occlusion*.

By occlusion is meant the arrangement of the cusps and sulci of the occluding surface of the artificial substitutes so as to be in proper relation to the antagonizing teeth, thereby restoring their normal functions. The arrangement should provide for correct position not only when the teeth are in direct occlusion, but also in their articulation or the act of bringing them into occlusion. A casual glance at a perfect natural occlusion of the human teeth will not be amiss here. In the first place there is an ideal arrangement for mutual support in all the teeth; the forms, sizes, arrangement of cusps and sulci, position of teeth in the arches, in short, everything tends to give to each the greatest possible support in all directions.

With but two exceptions each tooth has two antagonists, and the arrangement of these should be kept in mind, as should also the relations of the various marginal, oblique and triangular ridges, and also the mesial and distal inclined planes. The extension of the buccal and labial cusps of the upper over those of the lower and that of the lower linguals above the same of the upper, are also important facts to remember. Again the occlusal relation of the upper lingual cusps to the lower antagonizing teeth and the lower buccals to the uppers, should not be overlooked. A close study of these conditions cannot fail to impress one with the important place the teeth occupy in the maintaining of the arches in perfect relation and each tooth in perfect occlusion.

Keeping this in mind, are there any facts which warrant the assertion that the occluding surfaces of all crowns and bridges should be as near as possible to that of the natural teeth? In answer this paper will present two reasons only, which, in the opinion of the writer are sufficient, the first a theoretical and the second a practical

First. Proof as shown in comparative study of the teeth of animals. It is a well known fact that all animals, by a principle which is called "adaptive modification," will in time suppress those organs which are not used, and increase in development those which are. This principle is carried out in the development of the teeth.

In herbivorous animals for example, it is seen that the condyle of the mandible is comparatively large in area and the glenoid fossa is quite shallow, thus allowing wide and loose articulating movements; and the teeth which are used for grinding, are spread out and developed laterally to resist this transverse movement which is so necessary to the grinding of the refractory vegetable fibre. Their molar and premolar teeth are of the type called lophodont, the occluding surface of which presents peculiar longitudinal folds, which looks as if the enamel, dentine and cementum had been rolled together and cut off. The irregularity of the surface is due to the difference in densities of the constitutents of the teeth, the softer wearing the deeper. Now this arrangement of jaw and shape of teeth are inherited because the antecedants have, and these animals continue to eat, a special kind of food requiring the characteristic method of mastication.

Again in the carnivorous animals there are developed both a jaw movement and teeth whose functions are cutting and tearing, very little are no mastication proper taking place in the mouth. The condyle of the mandible is transversely elongated and the glenoid fossa is very deep. In consequence of this arrangement the mandible can perform an up and down movement only, any rotary or back and forward movement being impossible, this latter is further prevented by an extremely long coronoid process. The molars and premolars are generally more or less compressed and pointed; sometimes their crowns are flattened and slightly tuberculated, but they are never divided into lobes. In short, there is developed here an excellent machine for cutting, tearing and crushing.

In the study of the molars and premolars of man, the cusps are found to be raised into tubercules forming what is called the bunodont type. The cusps, crowns, roots and even the structural material of the teeth, have been developed because of the mixed diet of man, which requires both a vertical cutting as well as a horizontal grinding motion.

If now artificial substitutes be contsructed with smooth or slightly roughened occlusal surfaces, would it be at all surprising if the future generations of man be compelled like Nebuchadnezzar of old to live on grass? Is it not plain to all that God intended it in the course of nature that the cusps of the molars and premolars of man should have a certain and definite function to perform, and which for the good of humanity should be retained? Any failure on the part of the dentist to restore the natural contour in his work is therefore a degenerative step. Any perversion of the natural occlusion will form mal-occlusion and the latter continued is a great menace to the wellfare of the human race. Hundreds of dollars are being spent for the correction of mal-occlusion by the people, and needless time and worry by the dentists.

The Practical Proof.—A brief historical review of the upward development in the construction of artificial crowns will illustrate perfectly our profession's endeavors to imitate as nearly as possible the natural occlusion of the human teeth in artificial crown and bridge work.

The original gold crown was a simple cap, made by first adapting a band to fit the root, and then soldering a flat surface of gold on top of this band. Realizing the necessity of at least a roughened occlusal surface, small pieces of srcap gold were then soldered to the occlusal surface of the cap.

The inaccuracy of this occluding surface which decreased serviceability and usefulness led to the introduction of dies for stamping conventional caps with cusps.

The first productions in this line were individual dies obtained from natural teeth, being mounted upon a plaster base, carved to favorable shape for securing a mold in sand and a casting made in zinc.

A cusp was then swaged from plate gold into a discarded lead counter die usually by means of the die which nearest suited case in hand. This was so great an improvement and created such a demand that the supply houses manufactured these dies in sets of various numbers and made them of brass so as to be more indestructible.

The necessity for having a great variety of cusp forms in more compact shape led to the introduction of die plates, which was the next forward step. The first were plates containing counter-dies, into which the plate gold was swaged and the later forms contained dies over which the gold was adapted or impression taken and counter-dies made.

Following this there came the various systems giving a definite detailed scheme for the construction of crowns. These contained a great variety of tooth forms to meet the various occlusions. Some of these familiar to all are Hollingsworth's, Lowry's, Mellott's, etc.

The next step in advance was an improvement in the contour and occlusion. As shown in the systems which contain rubber tooth forms from which a counter-die was made of low fusing metal and a seamless crown obtained.

The approved method of to-day is seen in the use of the carved natural bite sectional and seamless crowns, which is the method advocated in this paper as the *only* scheme which approximates perfection in the occlusal contour.

For crowns the carved natural bite seamless is the best because a closer reproduction of the natural tooth form is possible and more satisfactory contour can be produced with nearly the same time consumed in the process as in the older schemes of making crowns.

The technic of this operation is probably familiar to all, briefly it consists in the construction of a primary copper band, which is adapted to root and bite taken by means of old modeling compound softened and placed into band while on root; after testing in different positions of articulation it is removed from mouth, sticky wax flowed into base in order to hold the compound in place, and the impression carved to resemble the natural tooth in same position, being careful not to destroy the bite. After carving is completed, a low-fusing metal counter-die is obtained from this tooth, by means of one of the various casting flasks; the three-part flask, or the one with the "practical" crown outfit, preferred. A seamless gold crown is then swaged into this mould.

An essential procedure in this operation is to trim the buccal and lingual aspects of the band sufficiently low as to be able to form the proper alignment of these two surfaces by carving.

Again it is absolutely necessary that the full occlusal contour should be restored, the lingual cusps of the lower molars, for example, should extend up and beyond the lingual cusps of the antagonizing upper molar. The occlusal surfaces should not diverge toward the lingual as is so frequently seen in crowns.

The same principle can be carried out in obtaining the occlusion of bridge work. If all gold dummies are to be used, the bite may be obtained by placing plaster or modelling compound between the mounted crown abutments on articulated models, dummies carved up, counter-dies obtained and then swaged in one piece preferably.

Or, if porcelain facings are to be used the occlusal surface of bite only is necessary. A counter-die of this is obtained and by the use of a long piece of plate, the entire occlusal surface is swaged in one piece, filled even full with solder, properly beveled and fitted to the facings.

In bridge work there is another vital point which is necessary to consider in connection with occlusion and that is stress. There is the peculiar construction of one or more dummies between two abutments, the latter receiving the full force of stress. According to an old principle "the uniting of two or more teeth causes such a modified and restrained motion of each tooth, that they can successfully withstand more force than the sum total of their individual resistances." This is, however, true in ideal circumstances only, where the long axis of the abutments are in parallel lines. But when they are not parrallel, when one abutment is receiving stress in the line of its greatest resistance, the other is receiving it at its least. In this latter condition, the force of occlusion should be lessened on the dummies and put in the line of greatest resistance on each individual abutment and it is important that the latter should be in proper bucco-lingual relation in order to avoid lateral motion. The technic of this can be no better carried out than by the scheme before suggested. The taking of bite in modeling compound or plaster gives the proper formation of cusps and by scraping and trimming same it is possible to increase or relieve stress wherever desired. The dummies should always be made smaller bucco-lingually than the crowns, in order to minimize the combined stress on the abutments.

An anatomical articulator is very necessary in all this work as the lateral motion is absolutely essential to properly articulate a bridge.

In porcelain crown and bridge work the proper occlusion and articulation of all pieces is by far the most important and essential point to be considered, for it not only increases usefulness as before suggested, but each individual tooth is subjected to less strain on account of stress being equally distributed over the entire occlusal portion of the bridge.

In conclusion everyone will acknowledge that if the profession is to continue the high standard it now enjoys, or better still, keeps on advancing, it will be necessary for us to study carefully the natural, that we may imitate more successfully.

Let us select for our goal as close an approximation as possible to that given by the donor of "every good and perfect gift," namely, "a perfectly natural artificial occlusion."

### DISCUSSION.

Dr. J. Q. Byram: I am glad, indeed, to hear a paper on crown and bridge work. It is said so often that everything in the profession goes in the line of paths, and since we have had our paths in crown and bridge work, the tendency now is to neglect the subject, and I wish to compliment Dr. Howell on the most excellent paper he has given us on this subject. If there is any branch of the profession that needs special attention, is is crown and bridge work. When I see the crown and bridge work that is done by myself and others I feel chagrined to think, we as professional men, pose as artists. We do not seem to have the first principles of art instilled into us, we are not only lacking from the artistic point of view, but we seem to lose sight too many times of proper mechanics. I assure you, it is a pleasure to hear this paper. I have contended for years that the only accurate way to attain occlusion for crown or bridge is to correct the lost articulation and construct a type for that particular case. While I use very often the seamless crown, I am not an advocate of it in any case where you supply the occluded surfaces. If it were common for us to find the teeth in normal condition or relations, I can see that we could use ideal methods in adapting our crown or bridge, but in ninety-nine cases out of a hundred we find that the tooth has been broken down until we have lost a certain amount of the space, and there is a moderate amount of extrusion of the antagonizing teeth, and as a result we have abnormal conditions and we cannot take any system which deals with the ideal and apply it to such a condition successfully. I think that this fact should be borne in mind more than it is.

I learned of a scheme last summer that has been of use to me. If you will take ordinary paraffine wax and melt it in a large spoon and stir plaster of Paris into it while it is in a molten condition, you will find that it will make one of the nicest carving materials that you have ever used. Make it plastic by heating and apply it to the primary crown and allow the patient to bite into it, and you have the cast.

I wish to lay particular stress upon the use of the anatomical articulator in crown and bridge work, and I wish to go one step further. In bridge work I advocate taking impressions of the entire mouth and articulating models of the entire mouth. You will find that it is seldom possible to take impressions of the half of the mouth and articulate them on an anatomical articulator and obtain the same accurate results that you can obtain if you have casts of the entire mouth, and I wish to emphasize that point, because it is neglected. I believe if we would use the anatomical articulator more in our crown and bridge work we would have fewer bridges loosening in a few years.

Dr. C. H. Worboys: There is one point that I am particular about in getting the occlusion in the mouth, and it was not mentioned in the paper. When you have the patient bite down on any material you may choose to use, don't forget to have him make a chewing action after your material has set well enough so that he can; until then hold it steadily. Let the jaws be moved and given the masticating motion while this material is in place, then you will be sure to have a prefect occlusion of the teeth in any point.

Dr. N. S. Hoff: The fact was brought out that it was necessary in making a piece of bridge work, and sometimes just as necessary in making a single crown, that you should have accurate articulation of the entire mouth to work with, unless you are going to work direct in the mouth itself, for the reason that it is impossible to get the proper masticating occlusion without having these models upon which to work. The idea of using these little crown articulators, representing perhaps only two or three of the chewing teeth, is almost as good as nothing at all; they practically amount to nothing. I believe that a great deal of our bridge work is destroyed because of the fact that it has not been properly made in reference to the direction in which the force is applied. The direction of the forces as applied have got to be studied and worked out on articulated models. A plaster impression of both jaws should be taken and accurately articulated on an anatomical articulator. They can't be articulated on a hinge articulator. I think it is of the utmost importance where anything like an extensive piece of bridge work is being constructed, that this should be done. It secures that kind of accuracy which will enable you to construct your bridge from a mechanical standpoint so as to resist the force of mastication and enable you to strengthen your bridges so that they will not break. I think this is one of the things that is sadly neglected and overlooked. If all these hinge crown articulators that are in existance were destroyed, it would be a good thing, as they are of no practical use except as expedients to hurry operations. The sooner we get out of that habit of hurrying things the better service we will render our patients.

Dr. Howell: In reference to what Dr. Byram said, of course, it is impossible to carry out what you call the line of occlusion at every point, but we can restore the occlusal contour. I meant that we could not do it with the ready-made dies.

